# Calcium Carbonate Prenucleation Cluster Pathway Observed via In Situ Small-Angle X-ray Scattering

**DOI:** 10.1021/acs.jpclett.2c03192

**Published:** 2023-05-09

**Authors:** Jonathan Avaro, Ellen M. Moon, Kai G. Schulz, Andrew L. Rose

**Affiliations:** † Southern Cross Geoscience, Southern Cross University, PO Box 157, Military Road, Lismore, NSW 2480, Australia; ‡ School of Engineering, Deakin University, 75 Pigdons Road, Waurn Ponds, VIC 3216, Australia; § Faculty of Science and Engineering, Southern Cross University, PO Box 157, Military Road, Lismore, NSW 2480, Australia

## Abstract

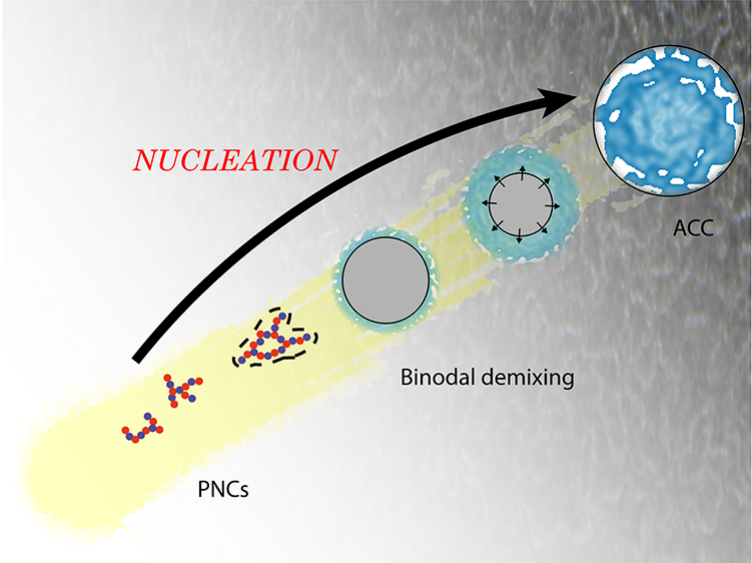

For more than 150 years, our understanding of solid-phase mineral formation from dissolved constituent ions in aqueous environments has been dominated by classical nucleation theory (CNT). However, an alternative paradigm known as non-classical nucleation theory (NCNT), characterized by the existence of thermodynamically stable and highly hydrated ionic “prenucleation clusters” (PNCs), is increasingly invoked to explain mineral nucleation, including the formation of calcium carbonate (CaCO_3_) minerals in aqueous conditions, which is important in a wide range of geological and biological systems. While the existence and role of PNCs in aqueous nucleation processes remain hotly debated, we show, using in situ small-angle X-ray scattering (SAXS), that nanometer-sized clusters are present in aqueous CaCO_3_ solutions ranging from thermodynamically under- to supersaturated conditions regarding all known mineral phases, thus demonstrating that CaCO_3_ mineral formation cannot be explained solely by CNT under the conditions examined.

Since first being proposed in the early 21st century,^1^ non-classical nucleation theory (NCNT) has engaged scientists concerned with organic, inorganic and protein chemistry and phenomena ranging from volcanic eruptions, meteorology, neurodegenerative disease, and even the origins of life.^2−,6^ In the prenucleation cluster (PNC) pathway for NCNT, PNCs are thought to act as the basic building blocks for mineral formation by subsequent aggregation and dehydration until a phase-separated solid is formed.^1^ This process may also involve a liquid–liquid phase separation step, which has been postulated to either result from the initial aggregation of highly hydrated PNCs or to occur prior to, and subsequently facilitate, PNC formation.^7−10^ Alternatively, it has been proposed that PNCs act as spectators only once the saturation state allows “true” homogeneous nucleation events to occur via CNT.^11−13^ In this scenario, PNCs do not play any role in nucleation and do not affect the nature of the future crystalline mineral phase. Therefore, there is a need to experimentally confirm both the existence of PNCs and the nature of their role in nucleation.

Investigations of potential aqueous CaCO_3_ (pre)nucleation processes have typically been performed at pH > 8.5 and/or under highly supersaturated conditions^14−17^ that are unrepresentative of most naturally occurring systems. Studies at low calcium or carbonate concentrations, corresponding to undersaturated conditions in which PNCs might be most readily observed and unambiguously identified according to the NCNT framework,^18^ have been hampered by the absence of experimental tools allowing sufficient sensitivity and resolution. Thus, the extent to which PNCs might influence aqueous CaCO_3_ nucleation at circumneutral pH values relevant to various biomineralisation and inorganic nucleation processes remains unclear. To overcome these challenges, we coupled a rapid mixing microfluidic device with in situ synchrotron-based SAXS, a widely used technique for direct observation of nanoparticle size and structure (Figure 1a). The recent establishment of a phase diagram for calcium carbonate phases delimiting the locus of spinodal and binodal demixing from which amorphous calcium carbonate forms was used as the basis for the establishment of saturation conditions in this work.^19^ Experimental conditions were chosen to cover the region around the liquid–liquid binodal demixing locus (aka amorphous solubility limit denoted here Ω_ACC_) and, thus, potentially identify via SAXS the formation of the dense phase’s precursors’ signature of NCNT. Nucleation was examined at two physiologically relevant pH values (pH 7.5 and 8.5, maintained using HEPES buffer, which has negligible binding affinity for Ca^20−22^) and a wide range of ion activity products (1.3 × 10^–9^ to 1.2 × 10^–6^ M), spanning conditions ranging from undersaturated with respect to calcite, the most thermodynamically stable CaCO_3_ mineral phase, to supersaturated with respect to much less thermodynamically stable amorphous calcium carbonate (ACC) phases (Extended Data Table 1).

**Figure 1:**
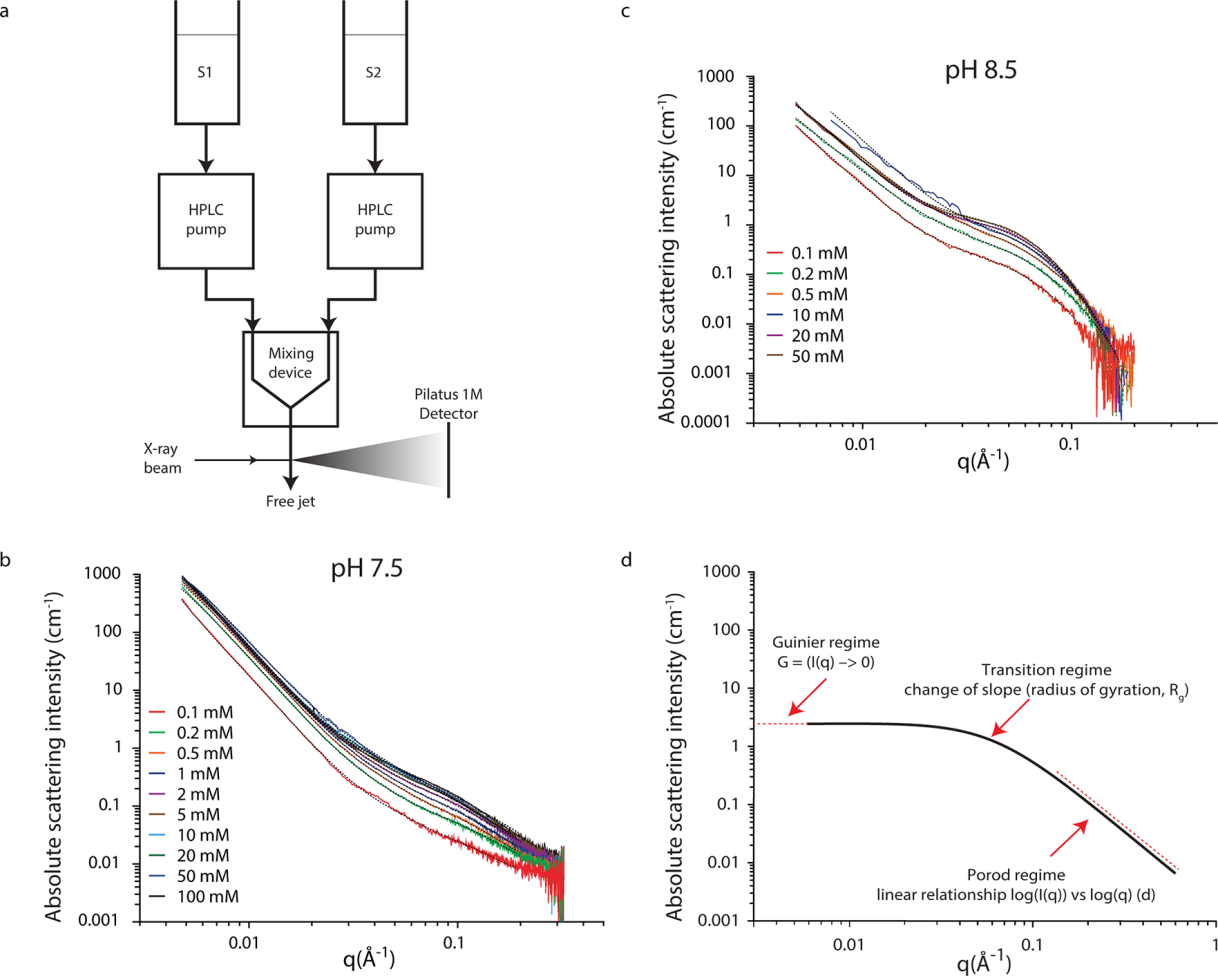
Observation of particle formation in undersaturated and supersaturated calcium carbonate solutions by SAXS. (a) Configuration of experimental apparatus. Solution S1 contained calcium chloride, and S2 contained sodium carbonate in HEPES buffer adjusted to pH 7.5 or 8.5. (b and c) Background-corrected X-ray scattering curves acquired at pH 7.5 and pH 8.5, respectively, for different total calcium concentrations (shown in legend). Dashed lines show the modeled scattering behavior, fit as the sum of scattering contributions from two independent structural levels described by the Unified Model (UM)^26^ (see Experimental Methods). Nonbackground subtracted data can be found in the Supporting Information. (d) Idealized curve describing scattering by a single population of scattering objects according to the UM.

Scattering data confirmed the presence of nanoparticles under all investigated conditions, exhibiting two distinct features: a hump in the scattering intensity *I* at values of the scattering vector *q* from 0.2 to 0.03 Å^–1^, corresponding to a particular population of nanoparticles, and a region at *q* < 0.03 Å^–1^ where *I* and *q* are related by a power law function indicative of larger scattering objects with a strong electron density gradient (sharp interface) toward the solvent (Figure 1b,c). The scattering feature at *q* < 0.03 Å^–1^ is consistent with idealized scattering from objects too large to be fully resolved over the *q* range examined. Similar features have consistently been observed in multiple previous SAXS studies of CaCO_3_ formation using a range of different experimental configurations (e.g., quartz capillary,^23^ quartz cuvette,^24^ and free jet^17,25^) and/or reaction time,^17,25^ implying this is unlikely to be an experimental artifact. Regardless, by deconvoluting the contributions from each of these features to the global scattering signal using a modified version of the unified model (UM),^26^ we removed the contribution of the feature at *q* < 0.03 Å^–1^ to focus on scattering due to the nanoparticles at *q* = 0.2–0.03 Å^–1^. This allowed characterization of two distinct types of nanoparticles with different size and structure under the two pH conditions examined (Figure 1d).

At pH 7.5, scattering data are indicative of particles with low structural dimensionality (e.g., planar or mass fractal structures) with a radius of gyration (*R*_g_) of 3.5 nm in the undersaturated domain with respect to calcite, later increasing from 3.2 to 5 nm in the undersaturated region with respect to ACC. Once the liquid–liquid binodal limit (Ω_ACC_) is crossed, this radius of gyration only increases from 5 to 5.5 nm. The structural domain dominating scattering at *q* = 0.03–0.2 Å^–1^ was characterized by a constant dimensionality parameter *d* ≈ 2, regardless of calcium concentration (Figure 2a). Values of *d* ≈ 2 are indicative of branched/planar/sheet-like or unfolded mass fractal morphology for monodisperse particles, but can also occur with highly polydisperse populations of objects with higher dimensionality (e.g., spherical morphology).^27^ Additional information is needed to differentiate between the two scenarios conclusively. Nevertheless, the dominance of bicarbonate ions at pH 7.5 and their propensity to act as chain terminators that limit cluster growth^14,16^ means substantial polydispersity seems less likely. The average *R*_g_ for these particles increased from 3 to 6 nm with increasing calcium concentration (Figure 2b). Analysis of the Porod invariant (Figure 3), a measure of total scattering power from a population of particles, further shows that the mean volume of each nanoparticle, *V*_P_, increased from 4 × 10^2^ to 1.4 × 10^3^ nm^3^ with increasing calcium concentration (Figure 3a). Regression of log *G* (where *G* is the asymptotic limit of *I* at low *q*, as obtained from the UM fit) against log *V*_P_ yielded a slope of ∼2 (Figure 3b), implying nanoparticle growth at pH 7.5 occurs via addition of monomeric units too small to be observed by SAXS (e.g., ions pairs or single ions) to existing particles.^28^

**Figure 2:**
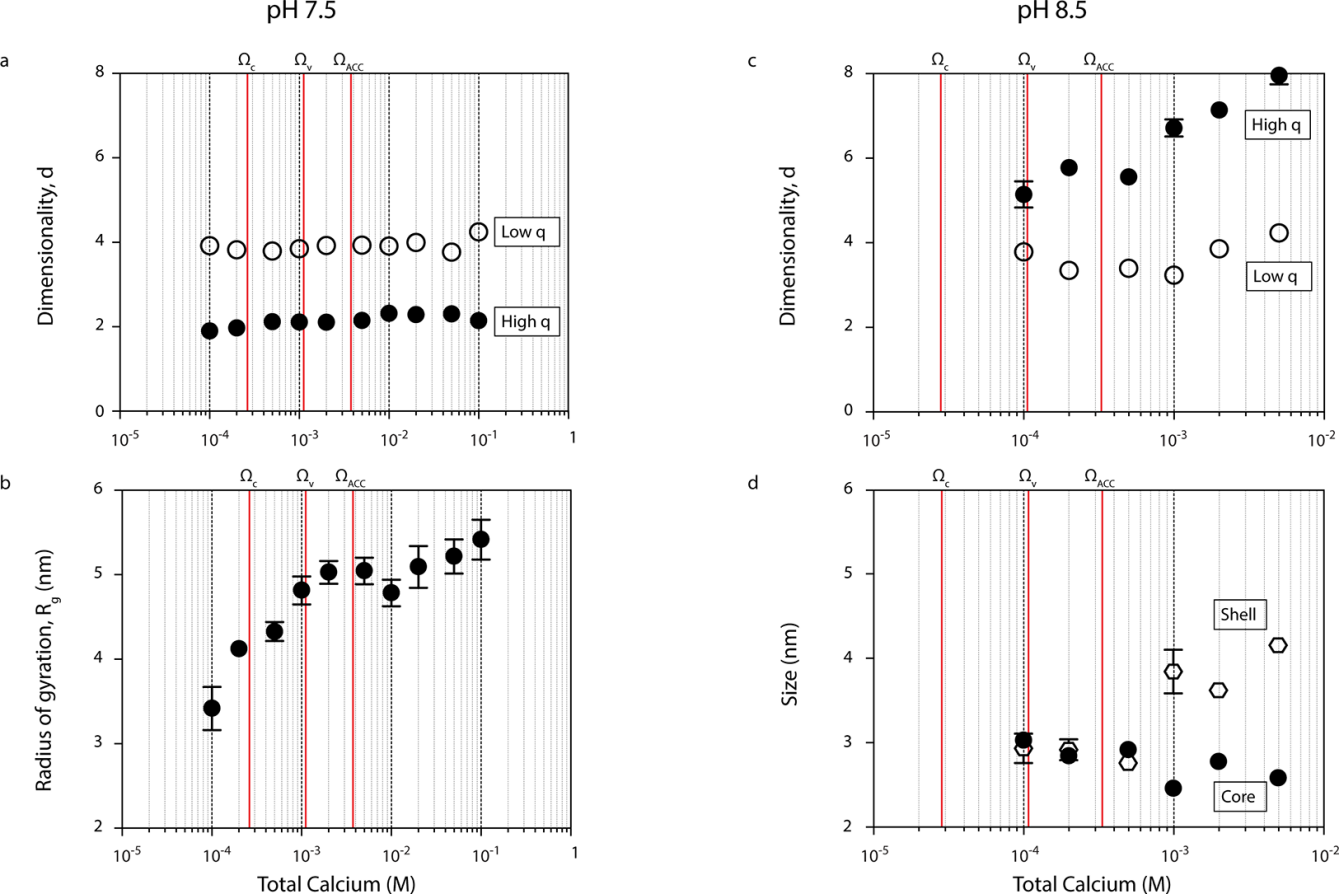
Size and structural characteristics of nanoparticles determined by the UM fit to SAXS data. (a) Dimensionality parameter *d* of high *q* and low *q* structural levels at pH 7.5. (b) *R*_g_ of high *q* scattering objects at pH 7.5. (c) Dimensionality parameter *d* of high *q* and low *q* structural levels at pH 8.5. (d) *R*_g_ of the spherical core (black circles) and thickness of the diffuse interface layer (white hexagons) of low *q* scattering objects at pH 8.5. Error bars represent the standard error from nonlinear regression fitting of the UM. Vertical red lines represent the solubility limits of calcite (Ω_C_), vaterite (Ω_V_), and amorphous calcium carbonate associated with the most stable liquid–liquid binodal demixing limit^19^ (Ω_ACC_). The diffuse interface thickness increases substantially when the binodal demixing limit—the solubility of ACC—is crossed.^9^

At pH 8.5, scattering data indicate spherical nanoparticles surrounded by a diffuse interface. The structural domain dominating scattering at *q* = 0.03–0.2 Å^–1^ was characterized by values of *d* > 4 (Figure 2c), implying the presence of a gentle gradient in electron density between the nominal surface of the scattering object and the solvent.^29^ The UM was modified to account for this by assuming the scattering objects could be represented by a spherical core with uniform electron density surrounded by a diffuse interface exhibiting sigmoidally varying electron density.^29^ Fitting the modified UM to the absolute scattering data enabled the calculation of *R*_g_ of the core and the thickness of the diffuse interface. *R*_g_ of the core decreased slightly from 3.0 to 2.5 nm while the thickness of the interface increased from 2.9 to 4.1 nm with increasing calcium concentration (Figure 2d). The overall size (*R*_g_ of the core plus thickness of the interface) thus rose from 5.9 to 6.6 nm with increasing calcium concentration. At pH 8.5, *V*_P_ remained constant at ∼2.7 × 10^3^ nm^3^ despite changing calcium concentration (Figure 3), so the particle formation mechanism could not be inferred from the approach used at pH 7.5.

**Figure 3:**
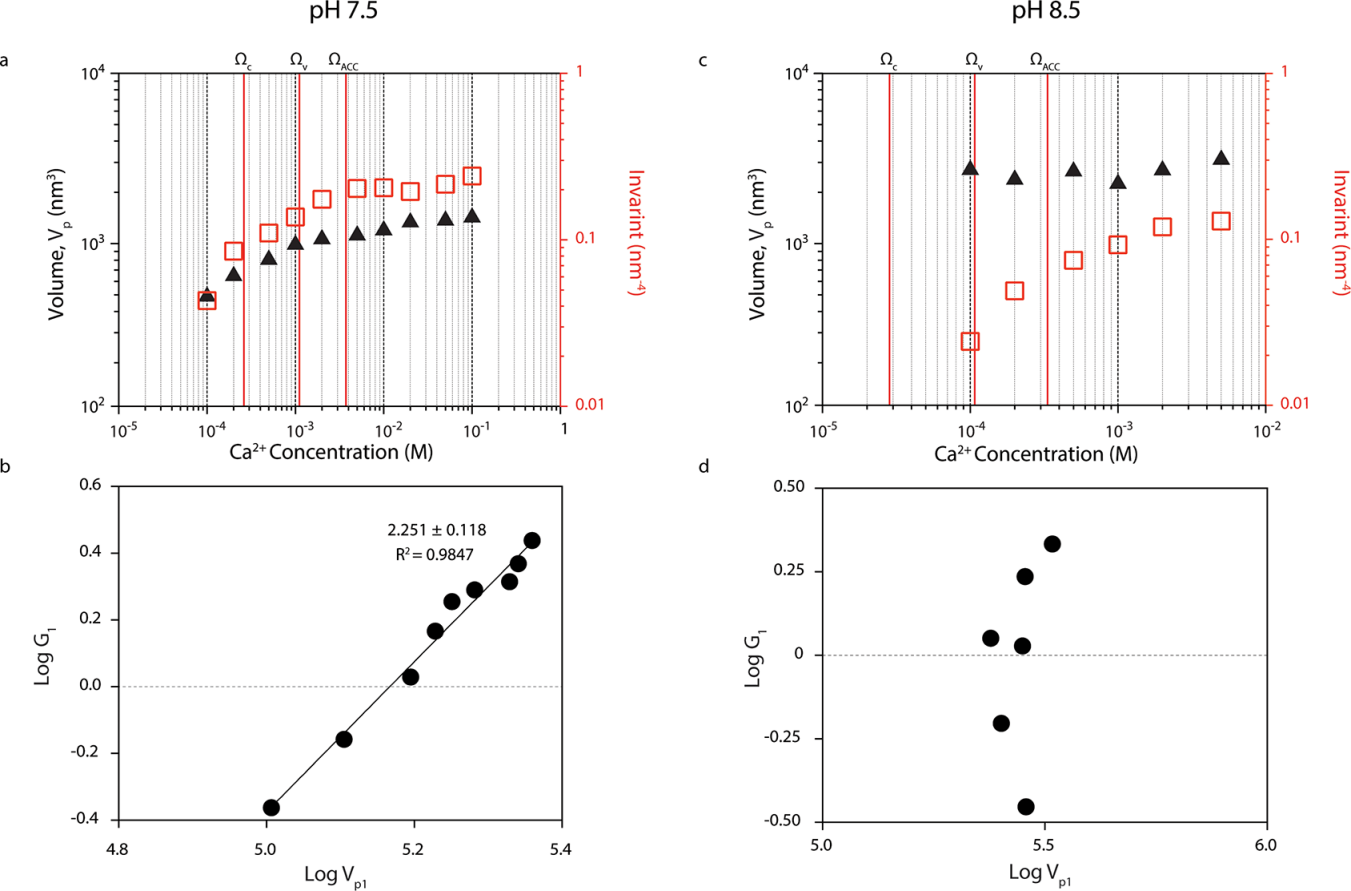
Nanoparticle volume and growth mechanism as determined from the Porod invariant. (a) Porod invariant (red squares; right axis) and average volume of nanoparticles (black triangles; left axis) corresponding to the structural level predominantly responsible for scattering at high *q* values (*n* = 1) (triangles) at pH 7.5. (b) Analysis of the growth mechanism of nanoparticles corresponding to the structural level predominantly responsible for scattering at high *q* values (*n* = 1) at pH 7.5. The linear relationship between log *V*_P1_ and log *G*_1_ with a slope of ∼2 indicates particle growth occurs via addition of single ions (or other clusters too small to be observed by SAXS) to the existing particle.^23,28,30^ (c) Porod invariant (red squares; right axis) and average volume of nanoparticles (black triangles; left axis) corresponding to the structural level predominantly responsible for scattering at high *q* values (*n* = 1) (triangles) at pH 8.5. (d) Analysis of the growth mechanism of nanoparticles corresponding to the structural level predominantly responsible for scattering at high *q* values (*n* = 1) at pH 8.5. A linear relationship between these parameters was not observed at pH 8.5, such that no information about the nanoparticle growth mechanism could be inferred. The vertical red lines in panels a and c represent Ω_C_, Ω_v_, and Ω_ACC_, the respective solubility limits of calcite, vaterite, and amorphous calcium carbonate, the latter associated with the most stable liquid–liquid binodal demixing limit^19^ (Extended Data Table 1).

The size and shape of the observed clusters in undersaturated conditions with respect to ACC are fundamentally inconsistent with CNT and its central concept of critical nuclei. In undersatured conditions, CNT describes the formation of transient, thermodynamically unstable clusters smaller than the critical nuclei that will form and quickly dissolve. Given that such clusters are very rare, transient, and extremely small species, it is impossible that they can explain the present scattering results.^1,6^ The particles observed here in conditions that are undersaturated with respect to all known CaCO_3_ polymorphs are at least an order of magnitude larger than ion pairs. These particles also possess a well-defined short-range order that sits, in terms of coordination environment, in-between the PNC structures obtained via computer simulation^14^ and any known CaCO_3_ polymorphs (Extended Data Figure 3),^31^ further confirming that they are not ion pairs, nor mineral phases resulting from local supersaturation due to imperfect mixing dynamics.

The size, shape, and structure of the observed clusters in supersaturated conditions, particularly at pH 8.5, and the “monomer-addition mechanism” of particle growth at pH 7.5 (Figure 3) are consistent with predictions from molecular dynamics simulations of nonclassical nucleation of aqueous PNCs.^14^ Furthermore, the progressive formation of a diffuse interface with increasing calcium concentration is consistent with molecular dynamics predictions of ongoing dehydration of the core under supersaturated conditions with respect to ACC (i.e., in the liquid–liquid binodal demixing regime^19^) (Figure 2d).^9^ The monotonic decrease in cluster size toward that of putative PNCs^1^ as calcium concentration decreases at pH 7.5 (Figure 2d) suggests the observed clusters represent a continuum from single PNCs to hydrated nanodroplet aggregates (Figure 3), consistent with the nonclassical aqueous CaCO_3_ nucleation mechanism proposed by Sebastiani et al.^9^ With increasing calcium concentration, the equilibrium between dissolved calcium ions and PNCs favors the formation of larger nanodroplets from PNC aggregation due to increasing cluster diffusion coefficient and/or bicarbonate acting as chain terminator.^14,16^ At higher pH and thus carbonate fraction, nanodroplets of hydrated CaCO_3_ formed in the liquid–liquid binodal demixing regime^19^ aggregate and progressively dehydrate (Figure 4). With increasing calcium concentration at pH 8.5, the size of the core decreases toward the predicted optimum for ACC stability^32^ of 3.8 nm diameter (equivalent to *R*_g_ of 1.5 nm for a sphere^33^).

These data provide compelling evidence that aqueous CaCO_3_ nucleation proceeds via the PNC pathway under the conditions examined and strongly suggest that PNCs participate in the nucleation process through a mechanism of liquid–liquid phase separation via aggregation of monomers (here, monomers being prenucleation clusters or any species smaller than our analytical detection window) followed by dehydration and densification of the droplets within the second liquid phase ultimately forming hydrated ACC (Figure 4). More importantly, the use of a mixing device and use of the shape-independent UM allowed us to acquire data of improved quality with regard to previous scattering studies on calcium carbonate (pre)nucleation systems.^17^ Observation of nanometer-sized clusters in undersaturated conditions provides direct evidence that more complex processes govern the prenucleation stage than the CNT describes and challenges findings of other recent studies^11,13^ which have described CaCO_3_ nucleation as the densification of a phase-separated liquid that forms an instant before nucleation and results in amorphous spherical objects 200–400 nm in diameter or again disproves the hypothesis of secondary nucleation as expressed elsewhere.^17^ While the limitations of these studies have been recently discussed,^34^ we emphasize the critical advantages of SAXS for observing coexisting hierarchical structures, such as resolving smaller systems embedded in larger particles as expected during nonclassical nucleation. Given the increasing body of evidence to support a nonclassical explanation of nucleation in a wide range of aqueous systems, there is a powerful incentive to re-evaluate previous nucleation studies through the lens of NCNT and consider more broadly the implications of CaCO_3_ PNCs for biomineralization and other globally important mineral formation processes.

**Figure 4:**
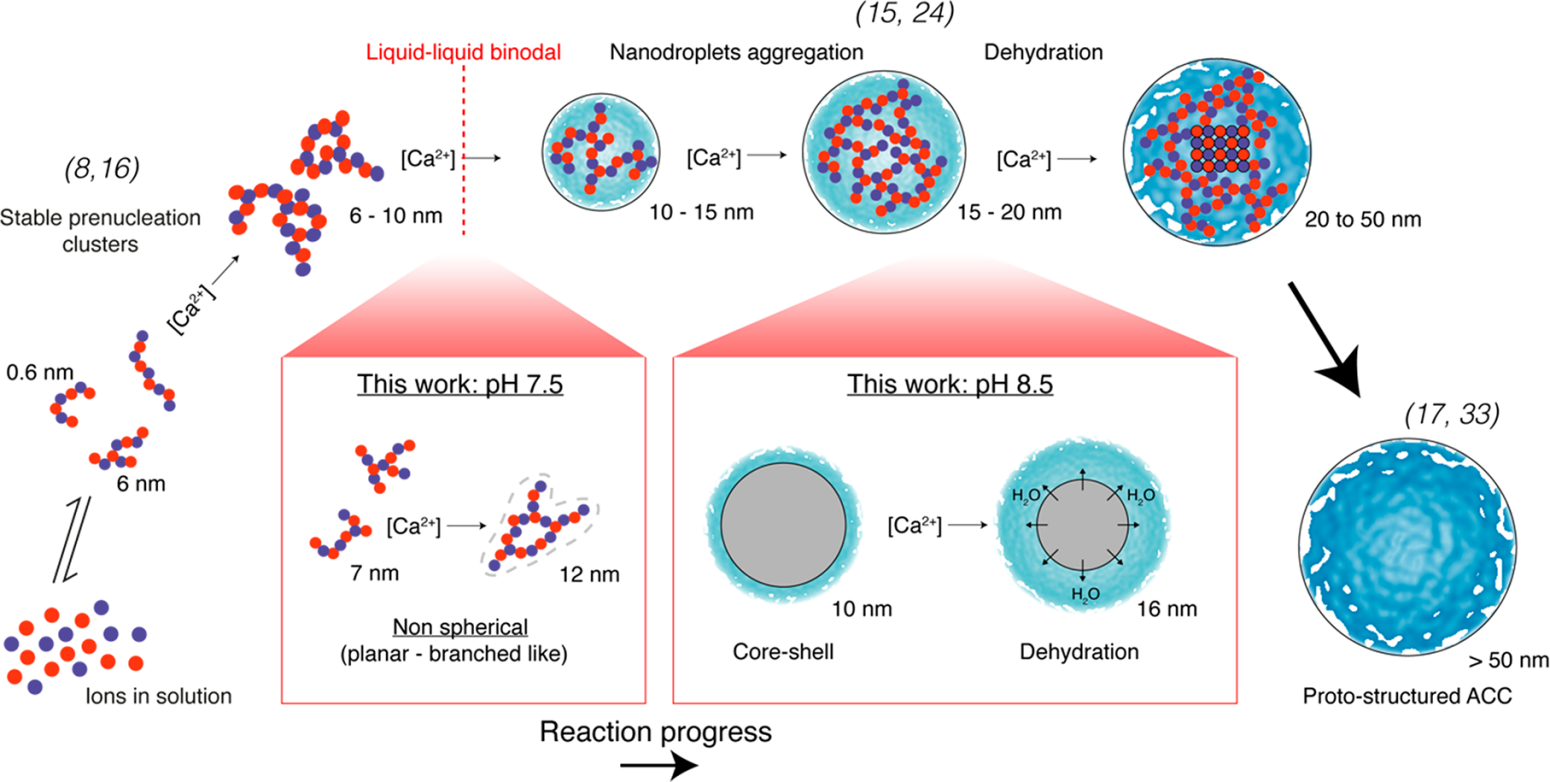
Schematic representation of the PNC pathway for ACC formation in aqueous solutions. Proto-structured ACC forms by the growth of PNCs into dense droplets that later aggregate, densify, and dehydrate. While the initial stages in the process are readily reversible, a critical transition occurs when the binodal limit for liquid–liquid demixing is crossed, leading irreversibly to the first phase-separated structures. The extent of reaction progress depends on a range of factors, including calcium concentration, total carbonate concentration, pH, and time. References to key studies relating to the various steps in the process are also shown. Adapted with permission from Sebastiani et al.^19^ Copyright 2017 Wiley-VCH Verlag GmbH & Co. KGaA.

## Experimental Methods

*Fabrication and Characterization of the Micromixing Device*. The mixing device was made from poly(methyl methacrylate).^31^ The device was designed in such a way that the fluid streams introduced through the supply channels are repeatedly divided into thin strata and recombined at very low Reynolds numbers, allowing mixing via diffusion on very short length scales and hence very short time scales. Under typical flow rates (a few mL·min^–1^), the residence time in the mixing chamber is close to 100 μs. The mixed flow is then convoluted due to the no-slip hydrodynamic boundary and surface topology, stretched, and accelerated in the exit channel to a higher Reynolds number (around 10–100), at which point inertia contributes to the formation of a free jet as the fluid exits the device. The mixing performance of the device was evaluated using the Villermaux-Dushman protocol.^31^

*Small-Angle X-ray Scattering (SAXS) Measurements*. SAXS measurements were performed at the SAXS/WAXS beamline at the Australian Synchrotron (Australia). Reagents were prepared using CaCl_2_·2H_2_O purchased from Ajax Finechem and Na_2_CO_3_ (assay ≥99.0%) and HEPES (4-(2-hydroxyethyl)-1-piperazineethanesulfonic acid) (assay ≥99.5%) purchased from Sigma-Aldrich. Stock solutions of 1 M CaCl_2_·2H_2_O and 20 mM Na_2_CO_3_ plus 20 mM HEPES were prepared in MQ water. HEPES was used as a noncalcium complexing pH buffer due to its fast response time and ability to maintain a quasi-steady-state pH after about 10 ms, which is appropriate for examination of calcium carbonate (pre)nucleation reactions under the conditions investigated in this study.

Measurements were performed by mixing a solution of CaCl_2_ (hereafter termed “reaction solution 3”) and a solution of 20 mM Na_2_CO_3_ and 20 mM HEPES buffer adjusted just before the beginning of the scattering experiment to pH 7.5 or 8.5 (hereafter termed “reaction solution 4”) in a 1:1 volume ratio using a continuous flow system (Figure 1a). Reaction solution 3 was bubbled with N_2_ for 30 min prior to commencing the experiment. The pH was measured using a glass pH electrode calibrated by a three-point curve with pH 4, 7, and 10 buffer solutions (pH 4 solution from Australian Scientific and pH 7 and 10 solutions from LabServ). The mixing device was operated at a total flow rate of 4.0 mL·min^–1^ (2.0 mL·min^–1^ for each of reaction solutions 3 and 4). The X-ray beam was focused as close as possible to the exit of the mixing device to obtain measurements at the shortest possible time after mixing (calculated to be 9.2 ms). One continuous run was conducted for each pH condition. The calcium concentration in reaction solution 3 was adjusted throughout each run by successive addition of calcium from a 1 M CaCl_2_ stock solution by a remotely operated syringe pump into a known volume of MQ water contained in a Schott bottle covered with Parafilm, which was continuously stirred via a magnetic stirrer. The calcium concentration in reaction solution 3 corresponding to each experimental condition was calculated by considering the initial volume of MQ water in the covered Schott bottle (220 mL), the amount of calcium stock solution delivered into the Schott bottle by the syringe pump at different times, and the rate at which the HPLC pump continuously withdrew the resulting solution from the Schott bottle (2.0 mL·min^–1^). Reaction solution 4 was stored in a Schott bottle covered with a Parafilm during experiments. Overall, 10 calcium concentrations were investigated ranging from 10^–4^ to 10^–1^ M, which was calculated to span approximately 1 order of magnitude below to 1 order of magnitude above the solubility limit of calcite at pH 7.5 and supersaturation with respect to calcite at all calcium concentrations at pH 8.5. Between each run, the whole system was rinsed with acidified MQ and pure MQ water in succession.

For each calcium concentration, 20 scans with an exposure time of 2.00 s each were acquired in “gapless mode” using an offset Pilatus 1 M solid-state detector and a camera length of 3100 mm, corresponding to a *q* range from 0.004 to 0.325 Å^–1^ at an incident X-ray energy of 12 keV. At the commencement of each run, an initial 20 scans were acquired before adding any calcium to reaction solution 3, then a further 20 scans were acquired for each calcium concentration. SAXS images were radially integrated to yield the scattering intensity curves *I*(*q*) as a function of the scattering vector *q* using the software ScatterBrain.^35^ For each condition, the 20 resulting curves were summed and background corrected by the subtraction of the average scattering intensity of the initial scans acquired prior to adding calcium to reaction solution 3. Data were calibrated using scattering from water as an absolute scattering standard and normalized by beamstop intensity to yield scattering intensity as an absolute value. Nonbackground subtracted scattering curves at different pH reflect the growth of nanoparticles with increase calcium concentration (Extended Data Figure 1). Background scattering data present at pH 8.5 a hump at ∼0.06 Å^–1^ not present at pH 7.5. To the best of our knowledge, we could not associate such a pH-dependent scattering feature to any carbonate or bicarbonate dissolved salts nor HEPES clustering. We acknowledge that under these conditions (10^–3^ μM/kg of dissolved inorganic carbon (DIC) and pH 8.5), the pCO_2_ equals ∼1050 μatm and would by consequence slightly outgas. However, the solution containing dissolved carbonate salts and HEPES buffer used as background and the fact that the scattering intensity increases with calcium concentration allow us to confidently say that increase of scattering intensity is linked to calcium-based nanostructures rather than outgassing of CO_2_ nanobubbles.

*Modeling of SAXS Data*. Small-angle scattering from polydisperse inorganic polymer systems often results in relatively featureless power law scattering on a log *I* vs log *q* plot, as observed here (Figure 1). Such scattering behavior can be modeled using a modified form of Beaucage’s unified model (UM), which is based on the principle that the overall scattering is composed of three associated regimes:

1. A Porod regime characterized by a linear relationship between log *I* and log *q* over a particular *q* range, where the negative of the slope (denoted *d*) relates to the dimensionality of the particles present;
2. A Guinier regime characterized by an asymptotic value of *I* at small *q* denoted as *G*, i.e.

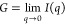

3. A transition regime characterized by an inflection point in the log *I* vs log *q* plot related to the *z*-weighted radius of gyration of the particles (denoted *R*_g_).

As the measured scattering curves are the sum of the scattering contribution from (potentially) multiple structural levels, each of these parameters can be fitted for any individual structural level, *n*. The UM expression for a particular structural level, as modified to account for Hammouda’s correction,^26^ is given by
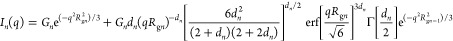
where *I*_*n*_(*q*) is the scattering intensity resulting from scattering by the *n*th structural level, erf is the error function, and Γ is the gamma function. Determination of *d*, *G* and *Rg* for each structural level (denoted by *d_n_*, *G_n_* and Rg_n_*R*_gn_ for structural level n) allows calculation of the contribution of each structural level to the overall scattering curve as follows: *I*(*q*) = ∑_*n*=1_^N^In(*q*) where *I*(*q*) is the overall scattering intensity and N is the total number of structural levels. Because of its empirical nature, the UM can be applied to any system exhibiting power law scattering regardless of the shape of individual particles and polydispersity of the particles, both of which influence the dimensionality value of the power law without changing the property of power law scattering. Fitting *d_n_*, *G_n_* and based on the UM thus allowed the reconstruction of the scattering curve resulting from each structural level over an infinite q range. Full details of the model fitting procedure are provided in Supporting Information.

## Supporting Information Available

Materials and Methods, Extended Data Table 1, and Extended Data Figures 1–3 (PDF)
Transparent Peer Review report available (PDF)
