# Supplementary material for: Calcium Carbonate Prenucleation Cluster Pathway Observed via In Situ Small-Angle X-ray Scattering
Source: J Phys Chem Lett. 2023 May 9;14(19):4517–23. doi: 10.1021/acs.jpclett.2c03192 (PMC10201568; doi:10.1021/acs.jpclett.2c03192)
Supplement: Supplementary file 1 — jz2c03192_si_001.pdf [file jz2c03192_si_001.pdf]

Supporting Information

Calcium Carbonate Prenucleation Cluster Pathway Observed via In Situ Small-Angle X-ray Scattering

Jonathan Avaro*, Ellen M. Moon, Kai G. Schulz, Andrew L. Rose

Correspondence to: jonathan.avaro@empa.ch

This PDF file includes:

Supplementary Materials and Methods
Extended Data Table 1
Extended Data Figures 1 to 3
Supplementary References

Supplementary Materials and Methods

Modelling of SAXS data.

Best fit parameter values for the UM fit to the experimental data were determined by non-linear least-squares fitting weighted by I^{-2} using GraphPad Prism. Only data between $q = 0.007$ Å^{-1} and $q = 0.16$ Å^{-1} were considered during modelling as anisotropy artefacts were present below $q = 0.007$ Å^{-1} (which were attributed to refraction of the X-ray beam from the side of the jet) and data at $q > 0.16$ Å^{-1} exhibited poor signal/noise ratio. Models with $N = 1$, 2 or 3 were considered but models with $N = 1$ and $N = 3$ failed to satisfactorily fit the scattering curves (as indicated by lower values of the weighted sum of squares for the model fit to the data, r^2, and the relatively high standard deviation of residuals). The presence of any additional structure levels can't be strictly excluded (as two structural levels presenting similarly close fitted parameters would be difficult to clearly distinguish), but statistically, the two fitted structural levels contributed to most of the measured scattering. Because the structural level corresponding to low q values ($n = 2$) did not exhibit a Guinier regime or transition regime, G_2 was arbitrarily set to 10,000 (i.e. the condition $G_2 >> G_1$ was enforced) and the value of R_{g2} was allowed to vary subject to the constraint that $R_{g2} >> R_{g1}$. The model was then used to fit d_2 independently of G_2 or R_{g2}.

Values of $d > 6$ observed under some conditions typically result from a diffuse interface between the scattering object and the solvent[1]. We considered this diffuse interface as a smooth evolution of electron density around a spherical scattering object as described by Guo et al.[2]. The electron density for a two phase system possessing a diffuse interface layer, $\rho_e(r)$, can then be expressed as the convolution of the electron density in an ideal two phase system separated by a sharp interface, $\rho_{e,id}(r)$, with a smoothing function $h(r)$, as follows:

$$\rho_e(r) = \rho_{e,id}(r) \otimes h(r) \quad (1)$$

where r is the distance along an arbitrary vector perpendicular to the boundary surface inside the scattering volume, $\otimes$ is the convolution operation and $h(r)$ the smoothing function[2]. The relationship between the scattering intensity of an object with a diffuse interface and that of an object with a sharp interface (i.e. an object obeying Porod's law with $d = 4$) is given by:

$$I(q) = I_{\mathrm{Porod}}(q) H^2(q) \quad (2)$$

where $I(q)$ is the scattering intensity function for the object with the diffuse interface, $I_{\mathrm{Porod}}(q)$ represents the scattering intensity function for an equivalent object with a sharp interface ($d = 4$) and $H(q)$ is the Fourier transform of the smoothing function $h(r)$. Assuming $h(r)$ is sigmoidal (follows a Gaussian distribution), is given by:

$$H(q) = e^{-(\sigma q)^2/2} \quad (3)$$

where σ is the standard deviation of the Gaussian distribution. The thickness of the diffuse interfacial layer, E, is then defined as:

$$E = 2\sqrt{3}\sigma \quad (4)$$

The UM was thus modified to incorporate this formulation as follows:

$$I_T(q) = I_1(q) e^{-(\sigma q)^2} + I_2(q) \quad (5)$$

where $I_T(q)$ is the total scattering intensity, $I_1(q)$ is the ideal Porod law scattering intensity produced by the uniform core of the structural level corresponding to high q values (*i.e.* $d_1 = 4$ for structural level $n = 1$) and $I_2(q)$ is the scattering intensity produced by the structural level corresponding to low q values ($n = 2$). This modified version of the UM was fitted to the data in the same way as the original UM, except that d_1 was set to a constant value of 4.

Different scattering models (such as different shape-dependent models, spinodal demixing models, or a correlation function) could have been used to represent the nature of the processes studied here, *i.e.* the formation of nanoscopic scattering objects, their growth via aggregation or monomeric addition processes, the formation of a liquid-liquid binodal demixing regime.

While those process described here seems close to the formation of a nano-emulsion with the formation of two phases within a continuous phase. The different scattering models for nano-emulsions ([3,4]) do not account for the key scattering features of this work, which are the size of the scattering objects with different overlapping regimes (scaling at low q-regime with the absence of a distinct Guinier regime). While the physical characteristics of a nano-emulsion via the formation of a phase-separated system are present in our system, the formation of this emulsion is not driven by the turbulence of the mixing device (where mixing is initially diffusion based)[5] but by the formation of droplets from the aggregation of hydrated PNCs slowly decreasing their dynamic.

Due to these differences and because we wanted to be able to model any and all scattering objects in a consistent manner, it was important to use a model that could account for changes in dimensional values, sizes, and aggregation processes that would reflect the physical phenomenon that might occur during the nucleation process. As such, we considered a shape-independent model to be the most appropriate, as this model type would allow us to describe a variety of scattering object types with a range of physical attributes through a bottom-up approach to the fitting that did not require making a priori assumptions about the shape or structure of the scattering objects. Choosing a model with less flexibility could have conferred unintended bias into our analyses, which given the contentious nature of this subject matter, we actively sought to avoid. The use a single multi-purpose model to fit all observed scattering objects is compatible with an objective and robust of process.

Calculation of particle volume and concentration. The total scattering power of each structural level, accounting for the scattering intensity from all possible geometries, is expressed by the Porod Invariant (Q_P), defined as:

$$Q_P = \int_0^\infty q^2 I(q)\,\mathrm{d}q = \phi_P(1-\phi_P)(\Delta\rho)^2 \quad (6)$$

where ϕ_P is the fraction of the sample volume occupied by scattering objects, and $\Delta\rho$ is the scattering length density of the objects in excess of that due to the background solvent (contrast)[6]. As the value of Q_P remains constant only over a particular q range (i.e. for a particular structural level), we can consider the evolution of the scattering power of each structural level individually[6,7] as follows:

$$Q_{Pn} = \int_0^\infty q^2 I(q)_n\,\mathrm{d}q = \phi_{Pn}(1-\phi_{Pn})(\Delta\rho_n)^2 \quad (7)$$

where ϕ_{Pn} is the fraction of the sample volume occupied by scattering objects corresponding to structural level n, and $\Delta\rho_n$ is the contrast of these objects.

For each experimental condition, Q_{Pn} was calculated for each structural level by integrating Kratky plots of $q^2I(q)_n$ vs q over an infinite q range, where $I(q)_n$ is given for a particular structural level (Figure 2). Q_P could only be calculated for the structural level corresponding to high q values ($n = 1$); the scattering curve for the structural level corresponding to low q values

(n = 2) could not be accurately reconstructed since G_2 and R_{g2} were not constrained by the UM fit in this region.

Calculated values of Q_P were then used to determine the mean particle volume V_{P1} (Figure 3), using the relationship[6]:

$$V_{P1} = \frac{2\pi^2 G_1}{Q_{P1}} \tag{8}$$

where values of G_1 are those obtained from the UM fit to the absolute scattering data.

In the case of a Smoluchowski-type cluster-cluster addition mechanism for particle formation, the ratio of log G / log Vp should be ~1, while this ratio should be ~2 for a monomer addition mechanism, where a monomer in this context represents any particle too small to be observed by SAXS in the q range examined[6,8] (Figure 3), subject to meeting several criteria relating to the properties of the particles[9].

Data availability. All primary data for this study are deposited in the Research Data Australia repository (https://researchdata.ands.org.au/).

Extended Data Table 1 | Solubility products for known calcium carbonate mineral phases in aqueous systems at temperature T = 294.15 K, salinity S = 0, and pressure = 1 atm. While the true salinity in our experiments is > 0, the values reported here represent lower limits as increasing values of S increase the solubility products of calcite and vaterite[10,11].

Mineral phase	K_{sp} (M^2)	Source
Calcite	3.48×10^{-9}	Calculated from the following equation in ref [10]: $\log K^{*}_{sp\,(Cal)} = -171.9065 - 0.077993T + \frac{2839.319}{T} + 71.595\log T + \left(-0.77712 + 0.0028426T + \frac{178.34}{T}\right)S^{0.5} - 0.07711S + 0.0041249S^{1.5}$
Aragonite	5.25×10^{-9}	Calculated from the following equation in ref [10]: $\log K^{*}_{sp\,(Ara)} = -171.945 - 0.077993T + \frac{2903.293}{T} + 71.595\log T + \left(-0.068393 + 0.0017276T + \frac{88.135}{T}\right)S^{0.5} - 0.10018S + 0.0059415S^{1.5}$
Vaterite	1.31×10^{-8}	Calculated from the following equation in ref [11]: $\log K^{*}_{sp\,(Vat)} = -172.1295 - 0.077993T + \frac{3074.688}{T} + 71.595\log T$
ACC Type I (proto-calcite)	3.1×10^{-8}	Ref [12] determined at 297.15±1 K with 10 mM total carbonate
ACC Type II (proto-vaterite)	3.8×10^{-8}	Ref [12] determined at 297.15±1 K with 10 mM total carbonate

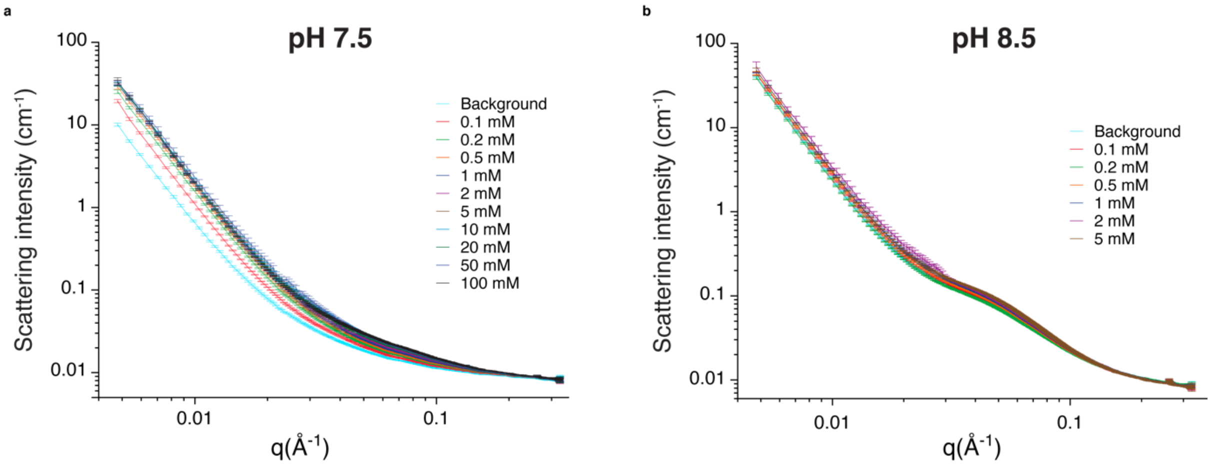

Extended Data Figure 1 | Non-background subtracted scattering data of calcium carbonate solutions. a, pH 7.5. **b,** pH 8.5. Carbonate solution containing HEPES pH buffer in milliQ water set at the desired pH condition was used as background.

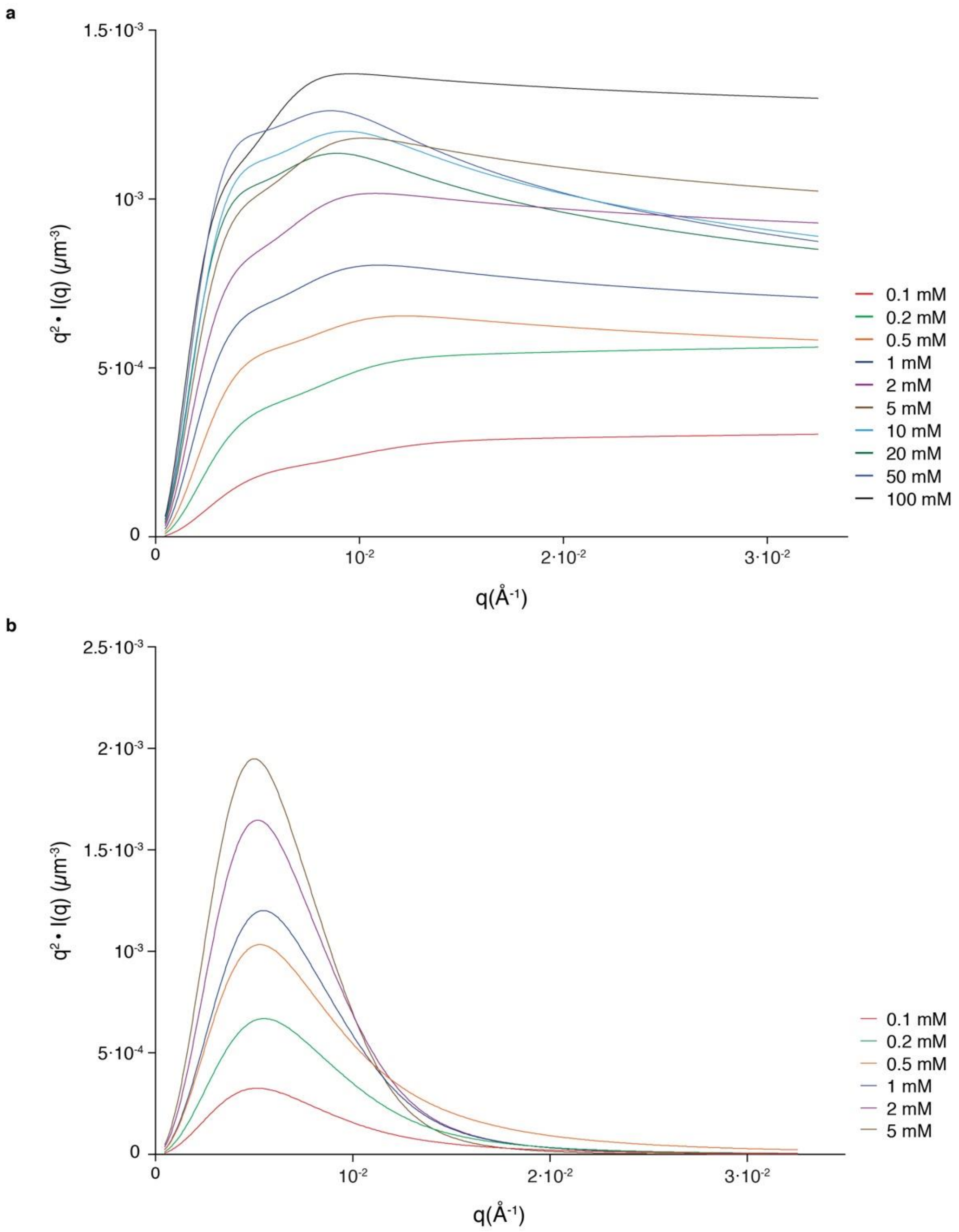

Extended Data Figure 2 | Kratky plots of scattering from the structural level corresponding to high q values (n = 1) over the range of calcium concentrations investigated (represented by different line colours as shown in the legend on each panel). a, pH 7.5. **b,** pH 8.5. Values of $q^2I(q)$ were calculated from the analytical function for $I(q)_1$ given by equation 5 using parameter values of d_1, R_{g1} and G_1 determined from the UM fit to the experimentally measured $I(q)$ values.

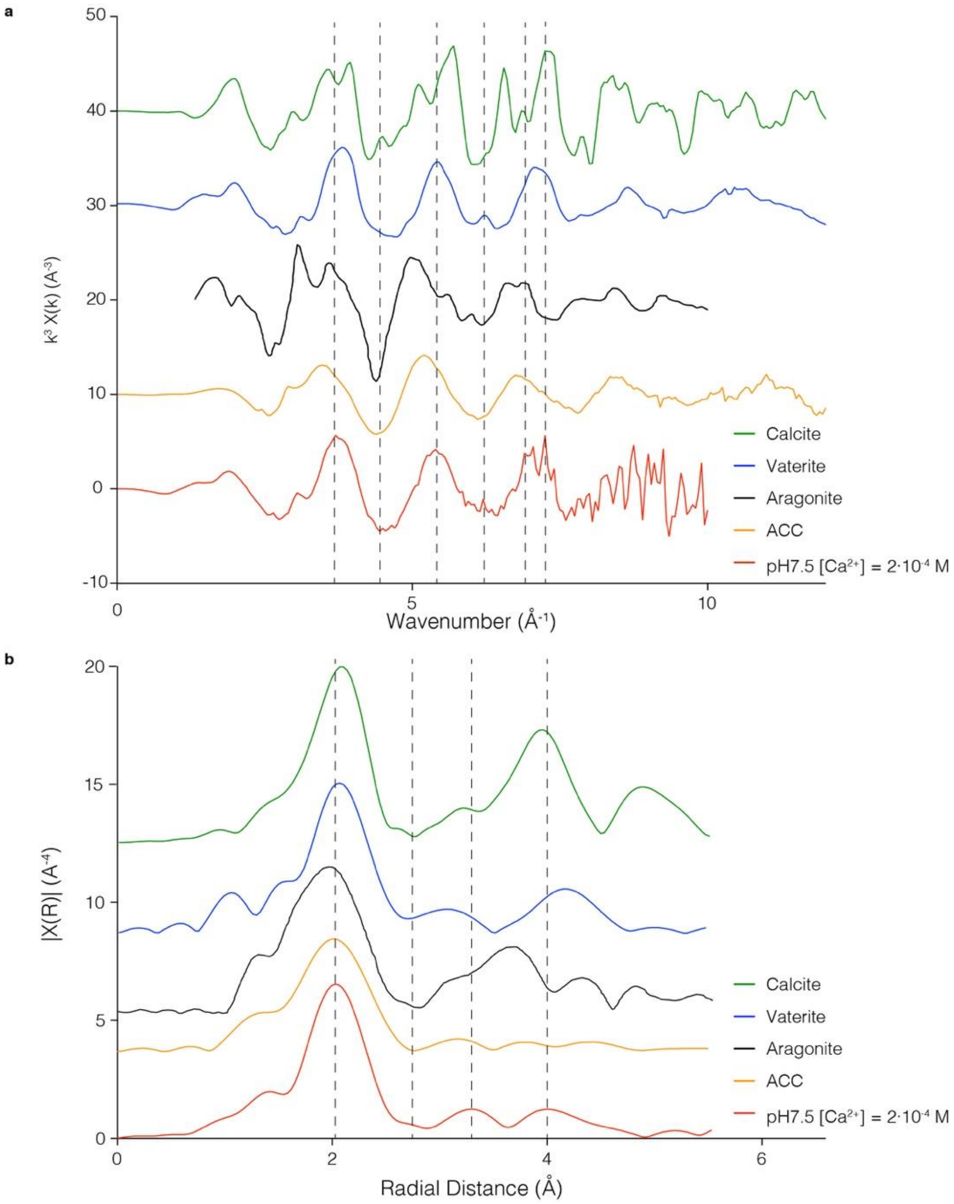

Extended Data Figure 3 | Background subtracted EXAFS spectra of $CaCO_3$ minerals and freeze-quenched samples containing 0.2 mM Ca and 10 mM total carbonate at pH 7.5. a, Ca K-edge EXAFS spectra. **b,** Fourier transform magnitudes. Green line = calcite, blue line = vaterite, black line = aragonite, orange line = ACC and red line = freeze-quenched sample. The spectrum for aragonite was not determined experimentally in this work, but instead reproduced from a previous report[13]. Dashed lines are used to guide the eye towards major features that differentiate the various spectra. The EXAFS spectra demonstrate that the freeze-quenched sample has a short-range order that is different from that of any known crystalline or amorphous polymorphs. While the spectrum of the freeze-dried sample prepared at pH 7.5 exhibits some similarities with the spectrum of vaterite, there is clear dephasing of several key features in *k*-space (panel a) and corresponding differences in the Fourier transform spectrum (panel b). All data are replotted from ref [14]. Full detail of data collection and analysis appears in ref [14].

Supplementary References

(1) Schmidt, P. W. Small-Angle Scattering Studies of Disordered, Porous and Fractal Systems. *Journal of Applied Crystallography* **1991**, *24* (pt 5), 414–435. https://doi.org/10.1107/S0021889891003400.

(2) Guo, X.; Gutsche, A.; Nirschl, H. SWAXS Investigations on Diffuse Boundary Nanostructures of Metallic Nanoparticles Synthesized by Electrical Discharges. *Journal of Nanoparticle Research* **2013**, *15*, 1–13. https://doi.org/10.1007/s11051-013-2058-7.

(3) Sheyfer, D.; Zhang, Q.; Lal, J.; Loeffler, T.; Dufresne, E. M.; Sandy, A. R.; Narayanan, S.; Sankaranarayanan, S. K. R. S.; Szczygiel, R.; Maj, P.; Soderholm, L.; Antonio, M. R.; Stephenson, G. B. Nanoscale Critical Phenomena in a Complex Fluid Studied by X-Ray Photon Correlation Spectroscopy. *Phys. Rev. Lett.* **2020**, *125* (12), 125504. https://doi.org/10.1103/PhysRevLett.125.125504.

(4) Servis, M. J.; Stephenson, G. B. Mesostructuring in Liquid–Liquid Extraction Organic Phases Originating from Critical Points. *J. Phys. Chem. Lett.* **2021**, *12* (24), 5807–5812. https://doi.org/10.1021/acs.jpclett.1c01429.

(5) Haberkorn, H.; Franke, D.; Frechen, T.; Goesele, W.; Rieger, J. Early Stages of Particle Formation in Precipitation Reactions - Quinacridone and Boehmite as Generic Examples. *Journal of Colloid and Interface Science* **2003**, *259* (1), 112–126. https://doi.org/10.1016/S0021-9797(03)00024-9.

(6) Rose, A. L.; Bligh, M. W.; Collins, R. N.; Waite, T. D. Resolving Early Stages of Homogeneous Iron(III) Oxyhydroxide Formation from Iron(III) Nitrate Solutions at PH 3 Using Time-Resolved SAXS. *Langmuir* **2014**, *30* (12), 3548–3556. https://doi.org/10.1021/la404712r.

(7) Beaucage, G. Small-Angle Scattering from Polymeric Mass Fractals of Arbitrary Mass-Fractal Dimension. *Journal of Applied Crystallography* **1996**, *29* (2), 134–146. https://doi.org/10.1107/S0021889895011605.

(8) Liu, J.; Pancera, S.; Boyko, V.; Shukla, A.; Narayanan, T.; Huber, K. Evaluation of the Particle Growth of Amorphous Calcium Carbonate in Water by Means of the Porod Invariant from SAXS. *Langmuir* **2010**, *26* (22), 17405–17412. https://doi.org/10.1021/la101888c.

(9) Liu, J.; Rieger, J.; Huber, K. Analysis of the Nucleation and Growth of Amorphous CaCO by Means of Time-Resolved Static Light Scattering Analysis of the Nucleation and Growth of Amorphous CaCO 3 by Means of Time-Resolved Static Light Scattering. *Langmuir* **2008**, *24* (13), 5123–5125. https://doi.org/10.1021/la8006519.

(10) Mucci, A. The Solubility of Calcite and Aragonite in Sea Water at Vaious Salinities, Temperatures and One Atmosphere Total Pressure. *American Journal of Science* **1983**, *283*, 780–799.

(11) Plummer, L. N.; Busenberg, E. The Solubilities of Calcite, Aragonite and Vaterite in CO2-H2O Solutions between 0 and 90°C, and an Evaluation of the Aqueous Model for the System CaCO3-CO2-H2O. *Geochimica et Cosmochimica Acta* **1982**, *46* (6), 1011–1040. https://doi.org/10.1016/0016-7037(82)90056-4.

(12) Avaro, J. T.; Wolf, S. L. P.; Hauser, K.; Gebauer, D. Stable Pre-Nucleation Calcium Carbonate Clusters Define Liquid- Liquid Phase Separation. *Angewandte Chemie* **2020**, *132* (15), 6212–6217. https://doi.org/10.1002/ange.201915350.

(13) Michel, F. M.; MacDonald, J.; Feng, J.; Phillips, B. L.; Ehm, L.; Tarabrella, C.; Parise, J. B.; Reeder, R. J. Structural Characteristics of Synthetic Amorphous Calcium Carbonate. *Chemistry of Materials* **2008**, *20* (14), 4720–4728. https://doi.org/10.1021/cm800324v.

(14) Avaro, J. T.; Moon, E. M.; Rose, J.; Rose, A. L. Calcium Coordination Environment in Precursor Species to Calcium Carbonate Mineral Formation. *Geochimica et Cosmochimica Acta* **2019**, *259*, 344–357. https://doi.org/10.1016/j.gca.2019.05.041.
