# Supplementary material for: Calcium Carbonate Prenucleation Cluster Pathway Observed via In Situ Small-Angle X-ray Scattering
Source: J Phys Chem Lett. 2023 May 9;14(19):4517–23. doi: 10.1021/acs.jpclett.2c03192 (PMC10201568; doi:10.1021/acs.jpclett.2c03192)
Supplement: Supplementary file 2 — jz2c03192_si_002.pdf [file jz2c03192_si_002.pdf]

jz-2022-03192r.R1

Name: Peer Review Information for "Calcium Carbonate Prenucleation Clusters Pathway Observed via In-Situ Small Angle X-Ray Scattering"

First Round of Reviewer Comments

Reviewer: 1

Comments to the Author

This is an excellent piece of work. Using a novel mixing device and in situ SAXS the authors demonstrate the formation of PNCs in the CaCO3-H2O system under conditions of pH and saturation never or poorly explored before. Their SAXS results, which have been analyzed using a novel modeling approach, confirm that PNCs form even in conditions of undersaturation with respect to calcite. This is a very important novel result demonstrating that PNC are indeed stable, even under such conditions, which suggests that they are widespread in many other relevant aqueous systems. The authors also show differences in PNCs size and shape/structure dependent on supersaturation and pH. In parallel, they show by means of EXAFS that the PNCs formed at pH 7.5 (relevant for physiological as well as natural earth surface conditions) do not have standard features (short-range order) corresponding to solids in the CaCO3-H2O system. In contrast, they show short-range order in between that of MD simulated PNC and known structures of CaCO3 polymorphs. Considering the novelty, relevance and broad implications of this study, I suggest acceptance for publication, provided the authors address the following issues:

1) One concern I have with this study is whether or not the observed SAXS features are due (at least in part) to the possible formation of HEPES nanoaggregates induced by the presence of Ca ions. While the authors detracted (as background) the contribution to scattering of the solution including HEPES buffer and Na2CO3, they did not performed control SAXS experiments using aqueous mixes of HEPES and Ca (dosed in the range of concentrations used here). Although they state that HEPES does not complexes Ca, and it is unlikely that it could cluster, I think they either need to provide further information (references) demonstrating that this is highly unlikely, or they might need to experimentally demonstrate that the features of their scattering curves are indeed solely due to the formation of PNCs, and not to the possible formation of nanoclusters of HEPES (following Ca-induced aggregation of the HEPES molecules).

2) In the Abstract and elsewhere in the main text the authors talk about "physiologically relevant conditions" which would apply for the biomineralization of CaCO3 (need to specify so). However, they also mention geological systems. It would be necessary to clarify what do they mean by physiological conditions in this latter case, as in many natural geological (inorganic) systems circumneutral pH conditions are clearly important, but in this case the term "physiological" does not seem to apply.

3) Lines 64-66: This sentence is not complete.

4) Figure 1b and 1c: The authors state in the caption: "Dashed lines show the modeled scattering behaviour, fit as the sum of scattering contributions from two independent structural levels described by the Unified Model (UM)". Honestly, it is very hard to see such fitting lines.

5) Lines 101-119: Here the authors describe the features of the SAXS curves for pH 7.5, in the case of the "unsdersaturated region with respect to ACC". I believe it would also be relevant to comment the specific features of the structures present in the undersaturated region with respect to calcite.

6) Lines 120-133: Why saturation conditions close to the solubility of calcite, or conditions of undersaturation with respect to this phase, were not explored? The lack of runs with Ca concentrations < 10-4 M precluded to get a more clear picture of the existence of PNCs at pH 8.5 in undersaturated conditions.

7) Line 144: "undersaturated conditions"… With respect to what phase?

8) Line 151: Replace "Extended Data Fig. 3" with "Extended Data Fig. 4". Check all references to "Extended Data Figs" as the numbering seems to be wrong thoroughout the main text and the Methods.

9) Lines 172-174: "PNCs participate in the nucleation process through a mechanism of liquid-liquid phase separation via aggregation of monomers…" Why monomers and not dimers or polymers? Note that if you claim that monomers contribute to the liquid-liquid phase separation, then you imply that PNCs, which by definition are not monomers, would not play a role in such a process. In any case, What is the experimental evidence showing that liquid-liquid phase separation occurs via aggregation of monomers?

10) Methods, Fabrication and characterization of the micro-mixing device: After the detailed description of the mixing device and the experiments performed to evaluate its mixing performance, the authors do not explain or justify why they selected a flow rate of 4 mL min-1 (corresponding to a mixing time of 9 ms), and not a higher flow rate, which should enable a smaller mixing time.

Reviewer: 2

Comments to the Author

As written, this manuscript describes a parametric fit of SAXS patterns with data-ranges and variables chosen to provide an outcome consistent with prenucleation theory. The text includes numerous examples of assumptions and exclusions favoring a PNC outcome. For example, dismissing the inability to distinguish between monodisperse or highly polydisperse particle populations (line 106) with the comment (line 112) “substantial polydispersity seems less likely”. Fitting the data from pH 7.5 to accommodate those from pH 8.5 requires modification of the UM model (line 133). Further discussion of fitting SAXS data (line 376+, lines 450-463) indicate further problems with the model and the necessity to assign parameters. The paragraph (line 144) is an unsubstantiated claim of inconsistency with CNT that ignores other available SAXS data on similar systems as well as information using other experimental techniques.

The authors make mention of the possibility of a liquid-liquid phase separation step in the introduction and then again in the Figure 3 caption. Their SAXS data provide intriguing evidence of this possibility.

The plot provided as Extended Data Figure 1, currently analyzed using the Porod approach, is particularly interesting. As mentioned in some of the references quoted in this manuscript, the interpretation of SAXS data in terms of critical phenomena and the Ornstein−Zernike relation may prove much more robust result than does their current Porod analysis. For example, SAXS has recently been used to argue the validity of utilizing a classical critical phenomena in complex, multicomponent solution-phase studies. References not mentioned, perhaps because of the apparently unrelated chemistry, including work in microemulsions (e.g. https://doi.org/10.1006/jcht.2002.0985) or on phase separation phenomena in chemical separations (e.g. https://doi.org/10.1021/acs.jpclett.1c01429 or https://doi.org/10.1103/PhysRevLett.125.125504). These studies often use T as the critical-field variable but critical phenomena theory has also used other field variables, including concentration ratios more pertinent to this case.

The close similarity of SAXS data provided in ED Figure 1 with those provided in Stephenson or other references related to structured solutions require, at minimum, a discussion of why their interpretation of SAXS data does not also pertain here. The relevance is that critical theory does not contain molecular-scale information but instead probes the macroscopic thermodynamic field variables and interparticle interactions driving phase transitions. From a more expansive perspective, a robust analysis with a more appropriate model is required to support the interpretation of SAXS data. Finding an alternate model may lessen the degree to which speculation and selective choice of parameters impact the outcome and may provide a more straightforward avenue to supporting the conclusions drawn in this manuscript.

The authors claim that their results “strongly suggest that PNCs participate in the nucleation process through a mechanism of liquid-liquid phase separation . . .”. This claim is not supported by the interpretation of the SAXS data on which they base this claim. Consequently, this manuscript is not recommended for publication in J. Phys. Chem. Lett.

Reviewer: 3

Comments to the Author

The authors present a highly compelling in-situ SAXS study of CaCO3 solutions under different pH conditions and concomitantly different saturation levels that are relevant to physiological mineral formation. The goal is to elucidate whether or not there exist pre nucleation clusters that would be characteristic of nonclassical nucleation theory. In fact, the authors do observe the formation of different characteristic pre nucleation clusters under these different conditions. This observation is significant to a wide range of the JPCL readership and would be large interest.

That being said - I admit I am not a SAXS expert however I do know that there is extensive literature that has shown that interpretation is not unique and that often several different structural models can fit the data. There is not much information in the literature associated with the analysis of the results, the approximations made and whether they are conclusive (i.e. can other models be omitted from the interpretation)? I would like to see more discussion of the data analysis and approximations made so that the conclusions could be more convincing given the limitations of SAXS. I also do not know the approximations made in the Unified Model.

Author's Response to Peer Review Comments:

Dear Editor,

We are pleased to share with you our answer to the three reviewers' comments on our publications. The changed text in the main article and the Supplementary material was highlighted in red to facilitate the review process.

We hope you will find these comments satisfying,

Best,

Reviewer(s)' Comments to Author:

Reviewer: 1

Recommendation: This paper is publishable subject to minor revisions noted. Further review is not needed.

Comments:

1) One concern I have with this study is whether or not the observed SAXS features are due (at least in part) to the possible formation of HEPES nanoaggregates induced by the presence of Ca ions. While the authors detracted (as background) the contribution to scattering of the solution including HEPES buffer and Na2CO3, they did not performed control SAXS experiments using aqueous mixes of HEPES and Ca (dosed in the range of concentrations used here). Although they state that HEPES does not complexes Ca, and it is unlikely that it could cluster, I think they either need to provide further information (references) demonstrating that this is highly unlikely, or they might need to experimentally demonstrate that the features of their scattering curves are indeed solely due to the formation of PNCs, and not to the possible formation of nanoclusters of HEPES (following Ca-induced aggregation of the HEPES molecules).

We thank the reviewer for this comment and have added additional text to lines 61 and 62 in the revised version of the paper along with supporting references (including recent work from Xiao et al, 2020) to clarify that HEPES buffer has negligible binding affinity for Ca.

2) In the Abstract and elsewhere in the main text the authors talk about "physiologically relevant conditions" which would apply for the biomineralization of CaCO3 (need to specify so). However, they also mention geological systems. It would be necessary to clarify what do they mean by physiological conditions in this latter case, as in many natural geological (inorganic) systems circumneutral pH conditions are clearly important, but in this case the term "physiological" does not seem to apply.

We have modified the abstact, removed the mention of physiological conditions, and modified the following sentence at lines 51 and 52 to clarify:
"Thus, the extent to which PNCs might influence aqueous $CaCO_3$ nucleation at circumneutral pH values relevant to various biomineralisation and inorganic nucleation processes remains unclear."

3) Lines 64-66: This sentence is not complete.

We have corrected this sentence. "The recent establishment of a phase diagram for calcium carbonate phase delimiting the locus of spinodal and binodal demixing from which amorphous calcium carbonate forms was used as the basis for the establishment of saturation conditions used in this work"

4) Figure 1b and 1c: The authors state in the caption: "Dashed lines show the modeled scattering behaviour, fit as the sum of scattering contributions from two independent structural levels described by the Unified Model (UM)". Honestly, it is very hard to see such fitting lines.

We have changed figure 1b and 1c, improving the visibility of the fitted lines.

5) Lines 101-119: Here the authors describe the features of the SAXS curves for pH 7.5, in the case of the "unsdersaturated region with respect to ACC". I believe it would also be relevant to comment the specific features of the structures present in the undersaturated region with respect to calcite.

We have added the phrase “[R_g] of 3.5 nm in the undersaturated domain with respect to calciate)to lines 93-94 to describe the lowest saturation point in our experiment. However, due to the linear evolution of the radius of gyration and constant value of dimensionality values between the points

undersaturated with respect to calcite to the saturation point of ACC, we do not see a direct interest in describing the first two saturation points in an independent manner.

6) Lines 120-133: Why saturation conditions close to the solubility of calcite, or conditions of undersaturation with respect to this phase, were not explored? The lack of runs with Ca concentrations < 10-4 M precluded to get a more clear picture of the existence of PNCs at pH 8.5 in undersaturated conditions.

We agree with reviewer one that after analysing the scattering data, it would have been highly interesting to look at the scattering processes occurring at pH 8.5 in the undersaturated domain with respect to ACC. However, this part of the phase diagram was not able to be explored in this study due to the method detection limit at low Ca concentrations. We also aimed to keep the total carbonate and calcium concentration comparable for the two pH conditions. An additional reason for limiting the investigation to such calcium concentration was due to having reasonable signal to noise ratio; lowering the concentration of calcium would have been shifting the equilibrium further toward unsaturated regime potentially generating detections limits. Such work could be the subject to a follow-up study.

7) Line 144: "undersaturated conditions"... With respect to what phase?

We have added a the phrase "with respect to ACC" to this line to describe the saturation state.

8) Line 151: Replace "Extended Data Fig. 3" with "Extended Data Fig. 4". Check all references to "Extended Data Figs" as the numbering seems to be wrong thoroughout the main text and the Methods.

We thank the reviewers for their comment and have updated the figure number and checked all the other reference entries.

9) Lines 172-174: "PNCs participate in the nucleation process through a mechanism of liquid-liquid phase separation via aggregation of monomers..." Why monomers and not dimers or polymers? Note that if you claim that monomers contribute to the liquid-liquid phase separation, then you imply that PNCs, which by definition are not monomers, would not play a role in such a process. In any case, What is the experimental evidence showing that liquid-liquid phase separation occurs via aggregation of monomers?

The authors agree with the reviewer's comments on the nature of the nucleation process as described in the nonclassical nucleation theory, where the liquid-liquid phase demixing process can occur via the aggregation of monomer, dimers and larger prenucleation clusters aggregate. In this study, the generic term monomer is relation to a “monomer addition” growth model, with monomer being a single ion or other dimers/clusters too small to be observed by SAXS, cf. coalescence processes. The experimental evidence is presented in the Extended Figure 3b of the Supplementary material. To avoid any confusion, we have added quotation marks around the term "monomer-addition mechanism" line 146, added the phrase “monomers being prenucleation clusters or any species smaller than our analytical detection window" in parentheses in lines 175-176, and added a reference to the Extended material Figure 3b.

10) Methods, Fabrication and characterization of the micro-mixing device: After the detailed description of the mixing device and the experiments performed to evaluate its mixing performance, the authors do not explain or justify why they selected a flow rate of 4 mL min-1 (corresponding to a mixing time of 9 ms), and not a higher flow rate, which should enable a smaller mixing time.

The mixing time of 4mL.min^{-1} was chosen to avoid any failure of the tube inserted in the mixing device during the length of the scattering experiment, which sometimes occurred at higher flow rates This explanation has now been added to the supporting information text.

Additional Questions:
Urgency: High

Significance: Top 10%

Novelty: High

Scholarly Presentation: Top 10%

Is the paper likely to interest a substantial number of physical chemists, not just specialists working in the authors' area of research?: Yes

Reviewer: 2

Recommendation: This paper is not recommended because it does not provide new physical insights.
We thank the reviewer for their comments, and have attempted to address the points raised by the reviewer in a constructive manner. We disagree, however, with the view that this paper does not provide any new physical insights, and note that this was clearly also not the view of the other reviewers.

Comments:

1) As written, this manuscript describes a parametric fit of SAXS patterns with data-ranges and variables chosen to provide an outcome consistent with prenucleation theory. The text includes numerous examples of assumptions and exclusions favoring a PNC outcome. For example, dismissing the inability to distinguish between monodisperse or highly polydisperse particle populations (line 106) with the comment (line 112) "substantial polydispersity seems less likely". Fitting the data from pH 7.5 to accommodate those from pH 8.5 requires modification of the UM model (line 133). Further discussion of fitting SAXS data (line 376+, lines 450-463) indicate further problems with the model and the necessity to assign parameters.

We respectfully disagree with the reviewer on this point. The “assumptions” that the reviewer refers to are based on what is already known about the nucleation behaviour of $CaCO_3$ under similar conditions and as such, are only assumptions in so much as previously published work may be shown to be incorrect at some point in the future, as is the case for any scientific work.

We took a bottom-up approach to fitting, using the so-called Unified Model primarily because it does not require a priori assumptions about the shape or structure of scattering objects, unlike most other methods. Furthermore, the parametric approach followed in this paper is not only based on the scattering model used but also accounts for the physical processes already known to occur during the nucleation processes of calcium carbonate based on previous studies – that is, the formation of PNCs in the undersaturated regime with respect to amorphous calcium carbonate phase and the liquid-liquid demixing processes ongoing past this point.

Based on the saturation ratios, one assumption that we have made is that the polydispersity of the scattering objects is rather low. The assumption was not made with PNCs in mind but based on the current understanding of undersaturated mineral regimes. As the reviewer mentions and as is noted in the manuscript, this is an assumption only in the sense that we have taken the findings of previous computer simulation published work (Demichelis at al 2011) to be valid and correct (see figure 5 with size of DOLLOPS), and we, therefore, consider this approach to be reasonable

We also wish to clarify that the modification to the UM used to fit the pH 8.5 data is an extension of the UM based on previously published work by other authors based on strong theoretical considerations – not simply additional parameterization to enable modelling of an otherwise impossible scenario. The existence of power-law scattering exponents > 4 occurs due to diffuse boundaries, as has been well described in the literature for several decades (see references in Guo et al. 2013, cited reference 35 in our paper), and this scenario is indeed described on p. 726 of the original paper expounding the formulation of the UM by Beaucage (1995) (doi: 10.1107/S0021889895005292). The modification we have used simply introduces an additional analytical relationship between the thickness of a diffuse interface between the scattering object and bulk solution and the UM power-law exponent, as described by Guo et al. (2013), which enables extraction of further information about the characteristics of such an interface. However, we emphasize that this is not "required" to "acccomodate" fitting the UM to the data at pH 8.5 – the same form of the UM used to fit the pH 7.5 works just fine at pH 8.5, but yields a power law exponent > 4 which is indicative of a diffuse interface.

However, we acknowledge that we can add further information to dispel concerns of bias towards a PNC outcome. We have added a paragraph in the supplementary information to describe the use of this model in this study compared to other available models.

2) The paragraph (line 144) is an unsubstantiated claim of inconsistency with CNT that ignores other available SAXS data on similar systems as well as information using other experimental techniques.

We would like to highlight the possible confusion regarding the nucleation behaviours between the super- and undersaturated regimes. In supersaturated regimes, the nucleation processes can indeed take the form to larger clusters than ion-pairs however, according the CNT in undersaturated regimes, only thermodynamically unstable small nuclei form and dissolve again and pre-critical nuclei are rare species that cannot result in the scattering as observed in this study. This is a critical point that not only supports our conclusions, but also supports the utility of the modelling approach taken as modelling approaches based on CNT would be unable to describe the observed data.

We have added additional information to describe the relationship between the scattering as observed and the concept of critical nuclei in lines 141 to 148 of the revised manuscript.

3) The authors make mention of the possibility of a liquid-liquid phase separation step in the introduction and then again in the Figure 3 caption. Their SAXS data provide intriguing evidence of this possibility. The plot provided as Extended Data Figure 1, currently analyzed using the Porod approach, is particularly interesting. As mentioned in some of the references quoted in this manuscript, the interpretation of SAXS data in terms of critical phenomena and the Ornstein–Zernike relation may prove much more robust result than does their current Porod analysis. For example, SAXS has recently been used to argue the validity of utilizing a classical critical phenomena in complex, multicomponent solution-phase studies. References not mentioned, perhaps because of the apparently unrelated chemistry, including work in microemulsions (e.g. https://doi.org/10.1006/jcht.2002.0985) or on phase separation phenomena in chemical separations (e.g. https://doi.org/10.1021/acs.jpclett.1c01429 or https://doi.org/10.1103/PhysRevLett.125.125504). These studies often use T as the critical-field variable but critical phenomena theory has also used other field variables, including concentration ratios more pertinent to this case.
The close similarity of SAXS data provided in ED Figure 1 with those provided in Stephenson or other references related to structured solutions require, at minimum, a discussion of why

their interpretation of SAXS data does not also pertain here. The relevance is that critical theory does not contain molecular-scale information but instead probes the macroscopic thermodynamic field variables and interparticle interactions driving phase transitions. From a more expansive perspective, a robust analysis with a more appropriate model is required to support the interpretation of SAXS data. Finding an alternate model may lessen the degree to which speculation and selective choice of parameters impact the outcome and may provide a more straightforward avenue to supporting the conclusions drawn in this manuscript.

The authors would like to thank the reviewer for their comments and information on the fitting processes of the phase separation. We refer the reviewer to the additional part of the supporting information (added in response to the reviewer's first comment), where we discuss the choice of the model and the limitation we have used for this work.

4) The authors claim that their results "strongly suggest that PNCs participate in the nucleation process through a mechanism of liquid-liquid phase separation . . .". This claim is not supported by the interpretation of the SAXS data on which they base this claimj. Consequently, this manuscript is not recommended for publication in J. Phys. Chem. Lett.

The authors would like to contest this comment by comparing the finding of these results with thermodynamic approaches as found by others [1,2]. Within the framework of this experiment, only the very early stage of the binodal demixing process is explored, and our fitting approach managed to probe the gradual dehydration of the nanoparticles, which to the best of our knowledge is a first for these conditions of (under)saturation, and is in good agreement with the expected physical behaviour observed via other methods.

(1) Sebastiani, F.; Wolf, S. L. P.; Born, B.; Luong, T. Q.; Coelfen, H.; Gebauer, D.; Havenith, M. Water Dynamics from THz Spectroscopy Reveal the Locus of a Liquid–Liquid Binodal Limit in Aqueous CaCO3 Solutions. *Angewandte Chemie - International Edition* **2017**, *56* (2), 490–495. https://doi.org/10.1002/anie.201610554.

(2) Avaro, J. T.; Wolf, S. L. P.; Hauser, K.; Gebauer, D. Stable Prenucleation Calcium Carbonate Clusters Define Liquid–Liquid Phase Separation. *Angew. Chem. Int. Ed.* **2020**, *59* (15), 6155–6159. https://doi.org/10.1002/anie.201915350.

Additional Questions:
Urgency: Moderate

Significance: Moderate

Novelty: Moderate

Scholarly Presentation: Moderate

Is the paper likely to interest a substantial number of physical chemists, not just specialists working in the authors' area of research?: No

Reviewer: 3

Recommendation: This paper is publishable subject to minor revisions noted. Further review is not needed.

Comments:
The authors present a highly compelling in-situ SAXS study of CaCO3 solutions under different pH conditions and concomitantly different saturation levels that are relevant to physiological mineral formation. The goal is to elucidate whether or not there exist pre nucleation clusters that would be characteristic of nonclassical nucleation theory. In fact, the authors do observe the formation of different characteristic pre nucleation clusters under these different conditions. This observation is significant to a wide range of the JPCL readership and would be large interest.

That being said - I admit I am not a SAXS expert however I do know that there is extensive literature that has shown that interpretation is not unique and that often several different structural models can fit the data. There is not much information in the literature associated with the analysis of the results, the approximations made and whether they are conclusive (i.e. can other models be omitted from the interpretation)? I would like to see more discussion of the data analysis and approximations made so that the conclusions could be more convincing given the limitations of SAXS. I also do not know the approximations made in the Unified Model.

We thank the reviewer for their comments. We have included in the Extended material a section called **Modelling of SAXS data** which includes an extended definition of the Unified Model used in this study and references for its approximations. We have added to this section a paragraph justifying the use of this model in this study compared to other available models.
"Due to the nature of the processes studied here - the formation of nanoscopic scattering object, their growth via aggregation or monomeric addition processes, the formation of a liquid-liquid binodal demixing regime – we want to acknowledge that different scattering models could have been used (such as different shape-dependent models, spinodal demixing model, correlation function). However, this study's special interest was ported to finding a unique and modulable way to describe the scattering objects observed via a shape-independent model to account for changes in dimensional values, sizes, and aggregation processes that would reflect the physical phenomenon expected during the nucleation process."

Additional Questions:
Urgency: Moderate

Significance: High

Novelty: High

Scholarly Presentation: High

Is the paper likely to interest a substantial number of physical chemists, not just specialists working in the authors' area of research?: Yes
